# Efficacy of chemo-immunotherapy in metastatic *BRAF*-mutated lung cancer: a single-center retrospective data

**DOI:** 10.3389/fonc.2024.1353491

**Published:** 2024-01-31

**Authors:** Ningning Yan, Huixian Zhang, Sanxing Guo, Ziheng Zhang, Yingchun Xu, Liang Xu, Xingya Li

**Affiliations:** ^1^ Department of Medical Oncology, The First Affiliated Hospital of Zhengzhou University, Zhengzhou, Henan, China; ^2^ Department of Medical Oncology, Shanghai Jiaotong University School of Medicine, Shanghai, China; ^3^ Prevention and Cure Center of Breast Disease, The Third Hospital of Nanchang City, Nanchang, Jiangxi, China

**Keywords:** immune checkpoint inhibitors, *BRAF*, NSCLC, programmed death-ligand 1, serine/threonine kinase

## Abstract

**Background:**

The effectiveness of combining immune checkpoint inhibitors (ICIs) with chemotherapy in treating non-small cell lung cancers (NSCLCs) with *BRAF* mutations has not been sufficiently explored.

**Methods:**

We compiled data from 306 NSCLC patients with identified *BRAF* mutations. We looked at efficacy by assessing the objective response rate (ORR) and disease control rate (DCR), as well as survival through measuring progression-free survival (PFS) and overall survival (OS).

**Results:**

Out of the patient pool, 44 were treated with a regimen of immune-chemotherapy. Patients undergoing ICI in combination with chemotherapy had a median PFS of 4 months, and the median OS was recorded at 29 months. There was a notable increase in OS in patients receiving first-line treatment versus subsequent lines (29 vs 9.75 months, p=0.01); however, this was not the case with PFS (9 vs 4 months, p=0.46). The ORR for patients on ICIs was 36.3%. PFS and OS rates did not significantly differ between patients with the *BRAF*-V600E mutation and those with non-V600E mutations (p=0.75 and p=0.97, respectively). Additionally, we found a significant variation in PD-L1 expression between those who responded to treatment and those who didn’t (p=0.04).

**Conclusion:**

Our findings indicate that chemo-immunotherapy as an initial treatment may lead to improved OS in patients with BRAF-mutated NSCLC when compared to its use in subsequent lines of therapy. Further studies are needed to validate these results and to delve deeper into how specific types of BRAF mutations and PD-L1 expression levels might predict a patient’s response to treatments in NSCLC.

## 1 Introduction

Treatment approaches for non-small cell lung cancer (NSCLC) have progressed significantly, advancing into an era of precision medicine. The latest developments include targeted therapies that are tailored to combat tumors that exhibit specific genetic alterations such as *EGFR* mutations, *ALK* and *ROS1* fusions, *BRAF* V600E mutations, *MET* exon 14 skipping mutations, *RET* fusions, *HER2* amplifications, and *NTRK* fusions. These innovative treatments have led to marked improvements in the overall survival (OS) rates for individuals battling advanced stages of NSCLC (1–8). Despite the high efficacy of these agents, the development of drug resistance is an inevitable hurdle that continues to pose a challenge in the management of the disease.

*BRAF* is a serine/threonine protein kinase found in the cytosol and encoded by the *BRAF* gene. This kinase is an important component of the *RAS* pathway, playing a central role in the regulation of various kinases including *MEK1/2* and *ERK1/2*, which are crucial for cellular signaling, proliferation, and survival (9–11). Although *BRAF* mutations contribute to continuous activation of the *BRAF* kinase, they occur relatively infrequently in NSCLC. Only about 2% of patients with lung adenocarcinoma have *BRAF* mutations, which are found more commonly in never-smokers, women, and those with more aggressive histological types like micropapillary patterns (12). *BRAF* mutations are categorized into three distinct types: *BRAF* V600E, known as class I or the classic mutation, and non-V600E mutations, comprising class II and III mutations. The *BRAF* V600E mutation is the most common and clinically actionable mutation found in NSCLC. Generally, this mutation is exclusive and does not occur alongside other oncogenic drivers. However, there are rare instances where *BRAF* mutations may co-occur with other mutations such as in the *KRAS* gene (13, 14).

Platinum-based doublet chemotherapy has had only moderate success in treating advanced NSCLC patients with *BRAF* V600E mutations. The objective response rates (ORR) for these patients have been low, and their survival outcomes have not been significantly improved by these platinum therapies (15–17). Studies indicate that NSCLC patients with *BRAF* mutations might see some benefit from *BRAF* inhibitor treatments (18–20). However, the response to *BRAF* inhibitor monotherapy in NSCLC with the *BRAF* V600E mutation has been less than optimal, with ORRs ranging between 33% and 42%, and a median progression-free survival (PFS) of only about 5.5 to 7.3 months (18–20). In contrast, the combination therapy targeting both *BRAF* and *MEK* inhibitors has shown more promise, achieving ORRs of about 63% to 64% and a median PFS of 9.7 months. When used as a first-line therapy, patients have experienced an increase in OS to an average of 24.6 months (21). However, these improvements have not been observed in patients with non-V600E *BRAF* mutations. Despite the potential of targeted *BRAF* inhibitors, their high cost and the scarcity of data from the Chinese patient population have limited their widespread adoption in China, making them inaccessible to most Chinese patients with *BRAF* V600E mutations. As a result, there’s an urgent need for more accessible and effective treatment options for NSCLC patients with *BRAF* mutations.

Immune checkpoint inhibitors (ICIs) have revolutionized the treatment strategy for NSCLC, especially for patients who lack driver gene mutations. However, there’s evidence to suggest that ICIs are less effective in NSCLC patients with *EGFR* or *ALK* mutations (22, 23). The efficacy of ICIs in patients with *BRAF*-mutant NSCLC is not well established, as some smaller studies indicate limited effectiveness when used as a monotherapy in this subgroup, with findings that have stirred some controversy (24, 25). A particular case report highlighted an NSCLC patient with a *BRAF* V600E mutation who experienced a prolonged beneficial response to a regimen combining ICIs and chemotherapy (26). This suggests that using ICIs together with chemotherapy might be a promising approach for treating *BRAF*-mutant NSCLC. Therefore, identifying the best initial systemic treatment for this patient population is still an ongoing endeavor.

In this study, we retrospectively analyzed the outcomes of 44 patients with advanced *BRAF*-mutant NSCLC who were treated with a mix of immune checkpoint inhibitors and chemotherapy. This analysis focused on assessing the effectiveness of the combined therapy by examining factors such as PD-L1 expression levels, tumor mutational burden (TMB), the line of treatment, and the specific type of *BRAF* mutation.

## 2 Patients and methods

### 2.1 Patients

We performed an extensive review of data from patients with advanced NSCLC who were treated at the First Affiliated Hospital of Zhengzhou University from July 2014 through December 2021. A total of 44 patients were evaluated for our study. We categorized them into two groups based on the type of *BRAF* mutation: those with V600E mutations formed Group A, and those with non-V600E mutations were in Group B. The inclusion criteria for the study were as follows: (1) age of 18 years or older; (2) a confirmed diagnosis of stage III/IV NSCLC through histopathological or cytological assessment, with an identified *BRAF* mutation; (3) patients who had undergone treatment that combined ICIs with chemotherapy; (4) at least one lesion measurable by the Response Evaluation Criteria in Solid Tumors (RECIST v1.1); and (5) previous radiation therapy, other treatments before joining the study, or recurrence post-radical surgery. We excluded individuals who had (1) received ICIs previously or (2) had early-stage tumors.The study protocols and procedures were granted approval by the Ethics Committee of the First Affiliated Hospital of Zhengzhou University.

### 2.2 Study design and data collection

To thoroughly investigate the treatment efficacy for advanced NSCLC, we engaged in an in-depth retrospective review and provided a flowchart to illustrate the study’s framework (see **Supplementary Figure 1** for details). Patient data were meticulously sourced from accurate and reliable medical records.

Our assessment of treatment response was routinely conducted at 6 to 8-week intervals by a pair of independent radiologists, using the RECIST version 1.1 guidelines. We compiled demographic information at the outset, which included sex (male vs. female), age (65 years and older vs. under 65), smoking history (never smoked, former or current smokers), line of treatment, and mutation status of genes. *BRAF* mutation status was determined utilizing RT-PCR or next-generation sequencing (NGS) techniques. The ORR was calculated based on the incidence of both complete responses (CR) and partial responses (PR). The disease control rate (DCR) spanned cases of CR, PR, and stable disease (SD). PFS was defined as the time from the initiation of ICI treatment to the onset of disease progression or the occurrence of death. For survival analysis purposes, patients were censored at their last follow-up appointment if they were alive without a progression of the disease. OS was measured from the beginning of treatment up until the death of the patients. In the survival analysis, patients were marked as censored if they were alive at their most recent follow-up visit.

PD-L1 expression levels were evaluated using immunohistochemistry (IHC) with a DAKO 22C3 PharmDx antibody. The accuracy and consistency of the PD-L1 IHC testing with the 22C3 PharmDx antibody have been well documented and are widely accepted by molecular pathology labs across Israel (27). The level of PD-L1 expression was measured using the tumor proportion score (TPS), which quantifies the percentage of tumor cells exhibiting partial or complete membrane staining. Based on the TPS results, patients were stratified into groups indicating negative (<1%), low (1%-49%), or high (≥50%) PD-L1 expression. Additionally, the TMB was examined via NGS, spanning an array of 425 genes according to the Foundation One sequencing platform, as has been outlined in prior research (28).

### 2.3 Statistical analysis

Statistical analyses were conducted using the most current version of IBM SPSS Statistics software, provided by IBM Corporation. For comparing associations between categorical variables, we utilized Fisher’s exact test and the Wilcoxon two-sample test. We adopted a two-sided hypothesis testing approach for all p-values, considering values below 0.05 as indicative of statistical significance. To estimate the median PFS and OS, we applied the Kaplan-Meier method, and survival curves were generated using the stratified log-rank test. Cox regression analysis was used to derive hazard ratios (HR) and their 95% confidence intervals (CIs).

## 3 Results

### 3.1 Patient demographic data

**Figure 1:**
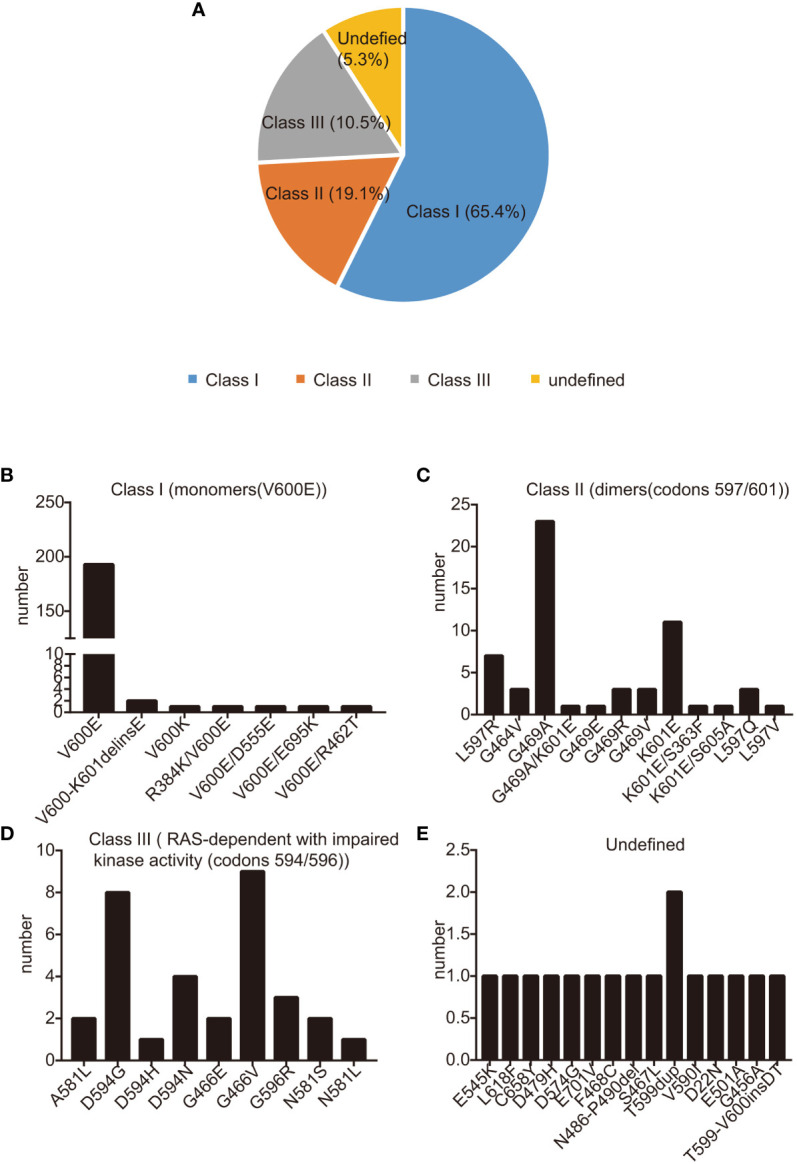
BRAF mutation categories. **(A)** BRAF mutations were divided into three classes: class I (65.1%), class II (19.1%), class III (10.5%), and Undefined (5.3%); **(B-D)** The occurrence rate of class I **(B)**, class II **(C)**, class III **(D)**, and undefined **(E)**: BRAF mutations in our study. Mutations are shown on the X-axis, while the Y−axis presents mutation frequencies.

Between July 1, 2014, and December 1, 2021, a total of 306 patients were diagnosed with *BRAF*-mutant NSCLC. Staging at diagnosis was as follows: 85 patients at stage I, 3 patients at stage II, 40 at stage III, and the largest group, 175 patients, at stage IV (refer to **Table 1** for details). Of those diagnosed, 28 patients (9.5%) presented with brain metastases at the time they were first found to have metastatic disease. The most common diagnosis was adenocarcinoma, representing 93.8% of cases, and a significant proportion of the cohort, 222 patients (72.5%), were either never-smokers or had a history of light smoking (less than 10 pack-years). The median age was 64 years, with a range spanning from 27 to 98 years, fitting with the clinical characteristics commonly seen in patients with *BRAF*-mutant NSCLC (16, 24, 29, 30). Similarly to past reports, we found that *KRAS* mutations are the most common co-occurring mutations with *BRAF*. In this cohort, 21 patients had both *KRAS* and *BRAF* mutations concurrently. Within this subgroup, 11 patients also had *EGFR* sensitizing mutations—including deletions in exon 19 and the L858R mutation in exon 21—in addition to their *BRAF* mutation. Notably, one patient had a concurrent *BRAF* mutation and *ALK* fusion, while another had *BRAF* mutation alongside a *CCDC6-RET* fusion (detailed in **Supplementary Table 1**). The methods used to detect the BRAF mutation varied, with the majority of mutations, 76.4% (234 patients), identified via NGS, and 22.2% (68 patients) detected through PCR. Both NGS and PCR methods were used for four patients (1.3%). The most commonly encountered mutation was *BRAF* V600E, found in 195 patients (63.7%). Other *BRAF* mutations, collectively termed non-V600E, such as G469A and K601E mutations, were also screened (illustrated in **Figure 1**). The specific treatment regimens and drugs administered as part of the study are listed in **Supplementary Table 2**.

**Table 1:** Patient demographic information.

Characteristics	Total BRAF-mutated (n=306)	Immunophenotyped cohort (n=262)	Treatment cohort (n=44)	p-value
**Median age (range), years**	66 (28–99)	66 (28–99)	65.5 (38–83)	0.95
**Sex** ** Male** ** Female**	162 (52.9) 144 (47.1)	136 (51.9) 126 (42.1)	26 (59.1) 18 (40.9)	0.67
**Smoking history** ** no** ** yes** ** Not specified**	222 (72.5) 81 (26.5) 3 (1.0)	191 (72.9) 68 (26.0) 3 (1.1)	31 (70.5) 13 (29.5) 0 (0)	0.94
**Histology** ** Adenocarcinoma** ** Squamous** ** Small cell** ** Adenosquamous** ** Sarcomatoid** ** Neuroendocrine** ** Large cell** ** Mucoepidermoid** ** Not specified**	287 (93.8) 7 (2.3) 1 (0.3) 5 (1.6) 2 (0.7) 1 (0.3) 1 (0.3) 1 (0.3) 1 (0.3)	247 (94.3) 3 (1.1) 1 (0.4) 5 (1.9) 2 (0.8) 1 (0.4) 1 (0.4) 1 (0.4) 1 (0.4)	40 (90.9) 4 (9.1) 0 (0) 0 (0) 0 (0) 0 (0) 0 (0) 0 (0) 0 (0)	0.71
**Stage** ** I** ** II** ** III** ** IV** ** NA**	85 (27.8) 3 (1.0) 40 (13.1) 175 (57.2) 3 (1.0)	85 (32.4) 3 (1.1) 35 (13.4) 136 (51.9) 3 (1.1)	0 (0) 0 (0) 5 (11.4) 39 (88.6) 0 (0)	0.002
**Brain metastases** ** Yes** ** No** ** NA**	28 (9.2) 184 (60.1) 94 (30.7)	22 (8.4) 148 (56.5) 92 (35.1)	6 (13.6) 36 (81.8) 2 (4.5)	0.002
**ICI combination therapy** ** First line** ** Second or later line **			27 17	

### 3.2 Immunophenotype

PD-L1 expression was assessed in 163 patients diagnosed with *BRAF*-mutant NSCLC, which included 43 from the treatment cohort and 120 with characterized immune profiles (detailed PD-L1 levels for each patient were depicted in **Supplementary Table 3**). The analysis showed that 89 patients (54.6%) had negative PD-L1 expression, 52 patients (31.9%) had an low level of expression, and 22 patients (13.5%) exhibited high PD-L1 expression (**Table 2**). TMB data were available for 38 patients with *BRAF* mutations; this subset comprised 11 patients from the treatment group and 27 from the immunophenotyped group. In this group, 28 patients had a TMB lower than 10 mutations per megabase (mut/Mb), accounting for 73.7%, while 10 patients (26.3%) had a TMB of 10 mut/Mb or more (**Table 2**). For a consistent comparative analysis, we focused exclusively on patients who had both NGS and TMB analyses conducted using the MSK-IMPACT panel, which is a broadly utilized genomic profiling platform. We calculated the median TMB in the lung tumors with *BRAF* mutations to be 6.3 mut/Mb, with values ranging from 0 up to 27.92 mut/Mb.

**Table 2:** PD-L1 expression and TMB status in BRAF-mutated patients.

	Total BRAF-mutated	Immunophenotyped cohort	ICI treatment cohort	*P*-value
**PD-L1 expression TPS (%)** ** Negative (<1)** ** low (1-49)** ** High (≥50)**	n=163 89 (54.6) 52 (31.9) 22 (14.5)	n=120 71 (59.2) 35 (29.2) 14 (11.7)	n=43 18 (41.9) 17 (39.5) 8 (18.6)	0.00
**PD-L1 expression TPS (%) mean**	17.22	15.04	25.78	0.21
**TMB (mut/Mb)** ** Low (<5)** ** Intermediate (5-10) ** ** High (≥10)**	n=38 17 (44.7) 11 (28.9) 10 (26.3)	n=27 13 (48.1) 13 (48.1) 1 (3.7)	n=11 4 (36.4) 6 (54.5) 1 (9.1)	0.09
**TMB (mut/Mb)** ** Median (range)**	7.3 (0–27.9)	0.7 (0–27.9)	9.2 (2.1–20.1)	0.1

### 3.3 Efficacy

**Figure 2:**
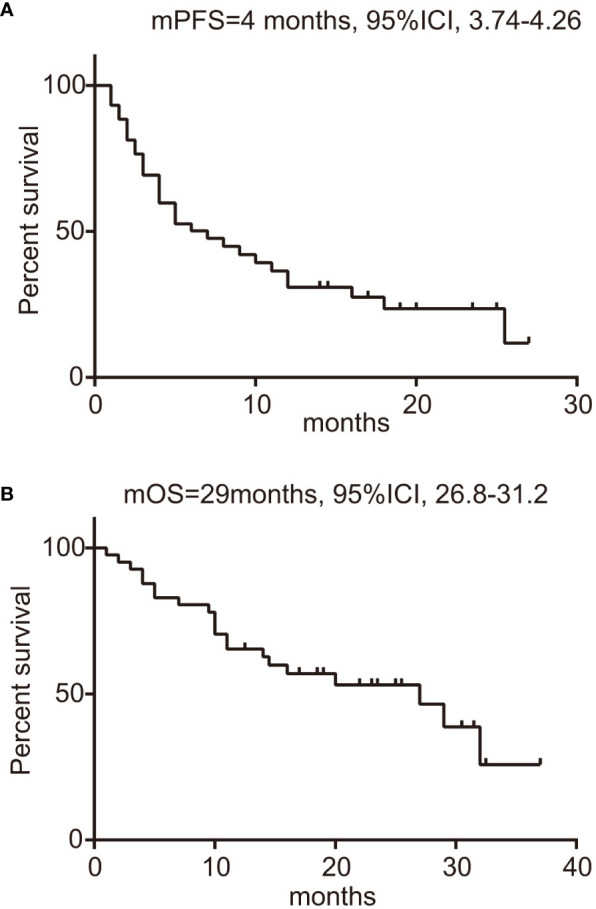
Survival analysis of BRAF-mutated patients treated with ICIs. **(A)** PFS of patients, with 95% confidence intervals indicated; **(B)** OS of patients, with 95% confidence intervals indicated. ICIs, immune checkpoint inhibitors; PFS, progression-free survival; OS, overall survival.

**Figure 3:**
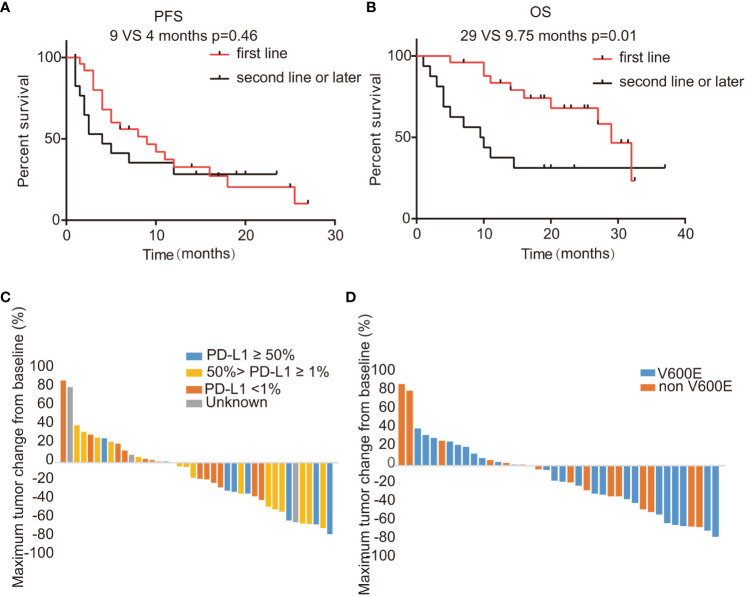
Activities of ICI combination regimens stratified by treatment lines, PD-L1 level, and BRAF mutation type. **(A, B)** PFS and OS for patients of the treatment cohort stratified by treatment line; **(C, D)** Changes in the sum of the longest diameters of target lesions based on different PD-L1 levels or BRAF mutation types. ICI, immune checkpoint inhibitor; PD-L1, programmed death-ligand 1; PFS, progression-free survival; OS, overall survival; mPFS, median progression-free survival; mOS, median overall survival.

In this study encompassing 44 patients treated with a regimen of ICIs combined with chemotherapy, we have detailed the workflow in **Supplementary Figure 1**. Within this cohort, 25 patients had combination therapy as their first-line treatment (post-progression therapy were summarized in **Supplementary Table 4**), while the remaining 19 patients received it in subsequent lines of treatment. By the data cutoff point, of the 15 patients with evaluable responses, we observed an ORR of 35.7% (95% CI: 20.6–50.8%), as shown in **Supplementary Table 5**. Specifically, within the first-line treatment group, the ORR was 40.7% (95% CI: 20.9–60.5%), which compares to an ORR of 29.4% (95% CI: 5.3–53.6%) among those treated in the second or later lines. This difference was not statistically significant (p=0.53), as depicted in **Supplementary Figure 2A** and **Supplementary Table 5**. The DCR was higher in the first-line group at 77.8% (95% CI: 61.0–94.5%) versus 58.8% (95% CI: 32.7–84.9%) in subsequent treatment lines (p=0.31), with further details available in **Supplementary Table 5** and **Supplementary Figure 2B**. We determined the median PFS to be 4 months (95% CI: 3.74-4.26) as illustrated in **Figure 2A**. The OS across all patients receiving ICI was recorded at 29 months (95% CI: 26.8-31.2), as shown in **Figure 2B**. Upon performing a subgroup analysis, we found that patients who received the combination therapies as their initial treatment had a longer median PFS when compared to those receiving the treatment in later lines (9 vs 4 months, p=0.46), which is represented in **Figure 3A**. Additionally, patients starting with the combination therapies in the first line of treatment displayed a notably greater median OS compared to patients who had the combination therapies in subsequent lines (29 vs 9.75 months, p=0.01), and this finding is presented in **Figure 3B**. Patients with *BRAF* V600E mutations are known to respond positively to *BRAF* targeted therapy, contrasting with those with non-V600E mutations. In our study, the ORR for those with V600E mutations was found to be 40% (95% CI: 19.4–60.6%), while the ORR for patients with non-V600E mutations was 31.6% (95% CI: 8.6–54.6%), as detailed in **Supplementary Figures 2C** and **2D**. Furthermore, the most significant changes in the diameters of target lesions, categorized by PD-L1 expression levels and BRAF mutation types, are illustrated in **Figure 3C, D**. The median PFS was 6 months for patients with V600E mutations and 8 months for those with non-V600E mutations, but the difference was not statistically significant (p=0.67, HR=0.75, 95% CI: 0.37–1.52), shown in **Supplementary Figure 3A**. Additionally, there was no observed difference in OS between the two mutation groups (p=0.97, HR=1.02, 95% CI: 0.41–2.53), presented in **Supplementary Figure 3B**.

### 3.4 Correlation of ICI efficacy with PD-L1 and TMB

**Figure 4:**
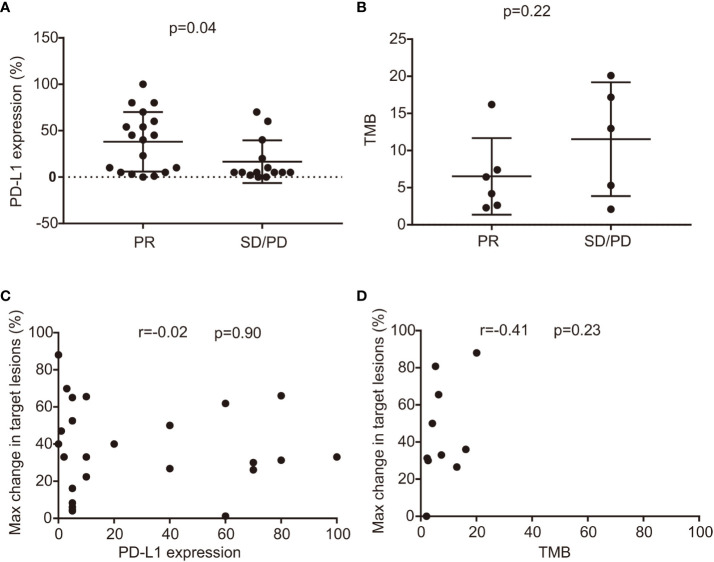
The correlation between efficacy, PD-L1 level, and TMB status. **(A)** relationship between PD-L1 expression and efficacy in responders (best response PR) and non-responders (best response SD and PD) (median 36% versus 18%, p=0.06); **(B)** Comparison of TMB in responders and non-responders treated with ICI combination therapies with TMB available (median 8.5 versus 7.3 mut/Mb, p=0.79); **(C)** The relationship between PD-L1 level and maximum changes in target lesions. **(D)** Correlation between TMB status and maximum changes in target lesions. CI, confidence interval; Max, maximum; mut/Mb, mutation per megabase; SD, stable disease; PD, progressive disease; PR, partial response; PD-L1, programmed death-ligand 1; TMB, tumor mutation burden; ICI, immune checkpoint inhibitor.

Past research has established a link between PD-L1 expression, TMB, and the effectiveness of ICIs (31, 32). In our study, we further explored these potential prognostic biomarkers in *BRAF*-mutant NSCLC. Our findings indicated a significant association between PD-L1 expression levels and responses to ICIs; specifically, responders (those achieving PR) had a median PD-L1 expression of 38%, comparing to 16% in non-responders (SD, or PD), with a p-value of 0.04 (as shown in **Figure 4A**). Conversely, no such correlation was observed when comparing TMB levels (median: 6.5 vs 11.5 mutations per megabase, mut/Mb, p=0.22), illustrated in **Figure 4B**. Moreover, patients with PD-L1 expression of 1% or higher experienced a longer PFS, averaging 9 months versus 4 months for those with less than 1% PD-L1 expression, although this result was not statistically significant (p=0.46) as depicted in **Supplementary Figure 4A**. However, a significant improvement in OS was noted; patients with 1% or more PD-L1 expression had a median OS of 29 months compared to 9.75 months for those under the 1% threshold (p=0.01) — this is presented in **Supplementary Figure 4B**. When comparing TMB to PFS or OS, there were no distinguishing differences (displayed in **Supplementary Figures 4C, D**). Similarly, no significant correlation materialized between the maximum reduction in the sum of target lesions and PD-L1 levels (r=-0.02, 95% CI: -0.42–0.37, p=0.90, represented in **Figure 4C**), or TMB (r=0.41, 95% CI: 0.29–0.83, p=0.23, as shown in **Figure 4D**).

### 3.5 Adverse events

Within the subset of patients treated with ICI combination therapies, 30 patients were documented with adverse events, with full details provided in **Supplementary Table 6**. For patients with *BRAF* mutations undergoing these treatments, the adverse effects were generally well-tolerated. The most frequently encountered adverse events were related to blood cell counts: neutropenia occurred in 16.6% of the patients, anemia in 36.7%, and thrombocytopenia in 14.3%. It’s noteworthy that serious instances (grade 3 or higher) of these hematological adverse events were quite rare, with 0% for neutropenia, 0% for anemia, and 3.3% for thrombocytopenia. Liver injury of a mild nature was seen in 30% of the patients, or nine individuals in total. Three patients experienced interstitial lung disease, prompting one patient to halt their treatment due to this issue. Additional side effects, including those affecting the gastrointestinal system, skin, and occurrences of hypothyroidism, are exhaustively listed in **Supplementary Table 6**.

## 4 Discussion

To our knowledge, this retrospective analysis represents the pioneering effort to investigate the immunophenotype and the impact of integrating immunotherapy with chemotherapy in Chinese patients with *BRAF*-mutant NSCLC. The findings suggest that these lung cancers might respond positively to what’s often termed chemo-immunotherapy, which was consistent with previous data from other regions (33, 34). However, due to the nature of a retrospective study and a somewhat small cohort of patients, we recommend additional studies to corroborate these initial results.

Previous research has suggested that PD-L1 expression tends to be higher in patients with *BRAF* mutations. Contrary to this, our study observed that the majority of *BRAF* mutation patients had low PD-L1 expression (≤ 50%), which appears to challenge these earlier reports (35–38). Retrospective studies with smaller cohorts have shown a pattern of positive PD-L1 expression in patients with *BRAF* mutations. For example, a study on 29 individuals with *BRAF* mutant NSCLC found that about 69% (20 out of 29) displayed positive PD-L1 expression, and over 40% had PD-L1 levels at or above 50%, indicating a considerable presence of PD-L1 positivity in this group (24). However, in our larger-scale study, we found that only 14.7% (22 out of 150) of patients had PD-L1 expression levels of 50% or more. The observed discrepancy could be related to our study’s larger sample size compared to earlier studies. Additionally, our data showed that most patients had a lower TMB, with a median value of 6.3 mutations per megabase (mut/Mb), which aligns with the notion that patients with *BRAF* mutant NSCLC may tend to have a lower or intermediate TMB and microsatellite stability (24). In our study, the majority of patients with *BRAF* mutations were found to be never-smokers, meaning they had either never smoked or had quit smoking more than twenty years ago. Previous research has indicated that a history of smoking is often associated with a higher TMB, which could explain the lower TMB we observed in *BRAF*-mutant NSCLC (39). Additionally, it’s been noted that a low TMB is a common feature in NSCLC patients with other types of oncogenic driver mutations (40–42). This might elucidate why patients with oncogenically driven cancers typically show only a moderate response to ICIs. Interestingly, our data revealed that some patients with BRAF mutations also carried other oncogenic drivers, such as *EGFR* mutations, *ALK*, and *RET* fusions. This contradicts the previously held belief that *BRAF* mutations are the exclusive driver of NSCLC in these patients. Despite encountering multiple oncogenic drivers, it’s important to highlight that concurrent mutations were relatively infrequent, with *BRAF* most commonly occurring alongside *KRAS* mutations.

Previous studies have shown that NSCLC patients with oncogenic driver mutations, such as sensitizing *EGFR* and *ALK* alterations, often see limited benefit from immunotherapy when used as a standalone treatment (22, 23, 43, 44). For example, research by Garon and colleagues revealed that untreated NSCLC patients with *EGFR* mutations who were treated with the ICI pembrolizumab had an ORR of 50% and a median PFS of 5.3 months. However, this contrasted sharply with the results of previously treated NSCLC patients with *EGFR* mutations, where the ORR dropped to 4% and the median PFS decreased to 1.9 months (45). Similarly, a comprehensive study on the effectiveness of ICI monotherapy in patients with NSCLC and *ALK*, *ROS1*, and *RET* fusions found that the most common outcome was disease progression. Thus, current clinical guidelines generally recommend against the use of ICI monotherapy as a subsequent line of treatment for NSCLC patients with these specific mutations. Comparable outcomes have been noted among patients with NSCLC who have *BRAF* mutations. Separate findings indicated that the response rate to ICI monotherapy in *BRAF* V600E mutant NSCLC patients was about 25%, with a median PFS of 3.7 months (24). Retrospective studies consistently report that ORRs for ICI monotherapy in *BRAF* mutant NSCLC patients vary between 10% to 30%, with median PFS times ranging from 2 to 4 months (22, 24, 25, 46, 47). Interestingly, the data also suggests that NSCLC patients with non-V600E *BRAF* mutations may respond better to ICIs than those with the V600E mutation. However, the overall survival tends to be longer in patients with the V600E mutation, possibly due to the effectiveness of targeted therapies against this specific *BRAF* mutation. As is the case with other oncogenic drivers, ICI monotherapy is typically not the treatment of choice for initial therapy in patients with *BRAF*-mutated NSCLC.

Our data indicate that combining ICIs with chemotherapy could be a promising treatment for patients with *BRAF*-mutant NSCLC. Despite an ORR of 36.3%, the treatment yielded a median PFS of 4 months and a median OS of 29 months, which are promising outcomes that warrant further investigation into this treatment combination. Supporting this, a case reported by Lu and colleagues detailed how a patient with *BRAF* V600E-mutant NSCLC responded well to a combined chemotherapy and immunotherapy regimen, achieving a PFS of 20 months (26). This case provides early evidence that patients with *BRAF* V600E mutations might benefit from a treatment strategy that includes both ICIs and chemotherapy. Moreover, the synergy of chemotherapy and immunotherapy has been demonstrated to improve survival rates for advanced NSCLC patients without driver mutations, a finding supported by results from previous randomized trials. These studies underscore the potential of chemo-immunotherapy as a significant advancement in the treatment of advanced NSCLC (48–52). Our results suggest that for patients with *BRAF*-mutant NSCLC, combination therapy involving ICIs may be a more favored approach compared to ICI monotherapy. It is essential to clarify, though, that this does not imply a blanket preference for ICI combinations over *BRAF* TKIs for all individuals with *BRAF* V600E mutations. Current treatment guidelines still prioritize *BRAF* TKI therapy for BRAF V600E-mutant NSCLC, guided by clinical trial data that indicated better survival outcomes (median PFS of 14.6 months with first-line *BRAF* TKIs; median PFS of 5–8.6 months with *BRAF* TKIs in subsequent lines of therapy) (4, 21). Additionally, previous real-world data found that patients with the *BRAF* V600E mutation achieved a 78% ORR, with a mOS similar to our findings. However, this study included only 17 evaluable patients, so the results should be interpreted with caution (53). Hence, treatment choices, including the potential use of ICI combinations or *BRAF* TKIs, should therefore be personalized, taking into account each patient’s unique profile and the specific line of therapy being considered. Additionally, in light of the considerable costs associated with *BRAF* TKIs, ICI combinations could be considered a viable alternative for patients with *BRAF*-mutated NSCLC, especially when dealing with later lines of therapy.

One notable finding was that only NSCLC patients with *BRAF* V600E mutations seem to benefit from *BRAF*-targeted therapies, while those with non-V600E *BRAF* mutations tend to respond less effectively to *BRAF* TKIs (18–20). Our study observed that patients with non-V600E *BRAF* mutations responded better to ICI combination therapy, achieving a median PFS of 10 months, in contrast to a PFS of 5 months for those with the *BRAF* V600E mutation. Although these results weren’t statistically significant, which may be attributed to the small sample size, they are suggestive of a potential trend. In addition, the ORR was higher in the non-V600E mutation group compared to the *BRAF* V600E group (40.0% vs. 31.6%), reinforcing the idea that non-V600E mutations might be more amenable to combination ICI therapies. This is in line with findings from other studies (22, 24, 46). Markedly, as NSCLC treatment becomes increasingly personalized, ICI combination treatments are becoming an important option for patients with non-V600E *BRAF* mutations. This treatment strategy might also be worth considering for later-line therapy in patients with the more common *BRAF* V600E mutation, illustrating the broader significance of ICI combinations in the treatment landscape of this heterogeneous disease.

Previous studies have indicated that PD-L1 expression and TMB might be effective biomarkers for forecasting responses to ICIs (31, 32). In line with these insights, our study found that PD-L1 expression was a reliable predictor of the success of ICI combination therapy in NSCLC patients with *BRAF* mutations. TMB, however, did not demonstrate a similar predictive ability. The absence of a correlation with TMB might be attributed to the relatively small number of patients in our study and the potential use of an unsuitable cutoff for TMB values.

Our research faces some significant limitations that warrant attention. To begin with, the small number of patients in our study could restrict the statistical strength and the ability to apply our findings more broadly. Additionally, not all study participants had PD-L1 levels and TMB evaluated, leading to potential variability and incomplete dataset issues. Moreover, the proportion of patients who underwent ICI therapy was limited, which narrows the breadth of our analysis. These factors, coupled with the retrospective design of the study and possible selection bias, advise a prudent approach in interpreting our results. Consequently, there’s a clear imperative for further prospective studies to confirm our observations and expand on the knowledge we’ve put forward.

Our findings indicate that NSCLC patients with *BRAF* mutations generally have lower PD-L1 expression and fall within the low to intermediate range for TMB. Treatments that combine ICIs with other therapies have shown promising results in controlling the disease and are well-tolerated by patients, whether they have *BRAF* V600E mutations or other types of *BRAF* mutations. Specifically, those with non-V600E *BRAF* mutations may experience even better outcomes from combination treatment regimens. Moreover, initiating treatment with these combination therapies could potentially improve the overall survival for those with *BRAF*-mutated NSCLC. These insights open new directions for research and underscore the potential for clinical innovations in treating this subset of NSCLC patients.

## Data availability statement

The original contributions presented in the study are included in the article/**Supplementary Material**. Further inquiries can be directed to the corresponding authors.

## Ethics statement

The present study and all protocols were approved by the Ethics Committee of the First Affiliated Hospital of Zhengzhou University on January 16th, 2023 (2022-KY-1516-002). The studies were conducted in accordance with the local legislation and institutional requirements. Due to the retrospective design and the sensitive nature of the individuals’ information, informed consent was exempted by the Ethics Committee of the First Affiliated Hospital of Zhengzhou University.

## Author contributions

NY: Conceptualization, Data curation, Formal analysis, Funding acquisition, Methodology, Project administration, Writing – original draft, Writing – review & editing. HZ: Conceptualization, Data curation, Formal analysis, Project administration, Resources, Software, Writing – original draft, Writing – review & editing. SG: Formal analysis, Methodology, Resources, Software, Writing – original draft, Writing – review & editing. ZZ: Data curation, Project administration, Resources, Writing – original draft, Writing – review & editing. YX: Data curation, Methodology, Writing – original draft, Writing – review & editing. LX: Conceptualization, Funding acquisition, Investigation, Project administration, Supervision, Validation, Visualization, Writing – original draft, Writing – review & editing. XL: Conceptualization, Investigation, Project administration, Resources, Supervision, Validation, Visualization, Writing – original draft, Writing – review & editing.
