# Supplementary material for: Efficacy of chemo-immunotherapy in metastatic BRAF-mutated lung cancer: a single-center retrospective data
Source: Front Oncol. 2024 Jan 31;14:1353491. doi: 10.3389/fonc.2024.1353491 (PMC10865094; doi:10.3389/fonc.2024.1353491)
Supplement: Supplementary file 1 [file DataSheet_1.docx]

**Supplementary Materials**

**Supplemental Figure 1**


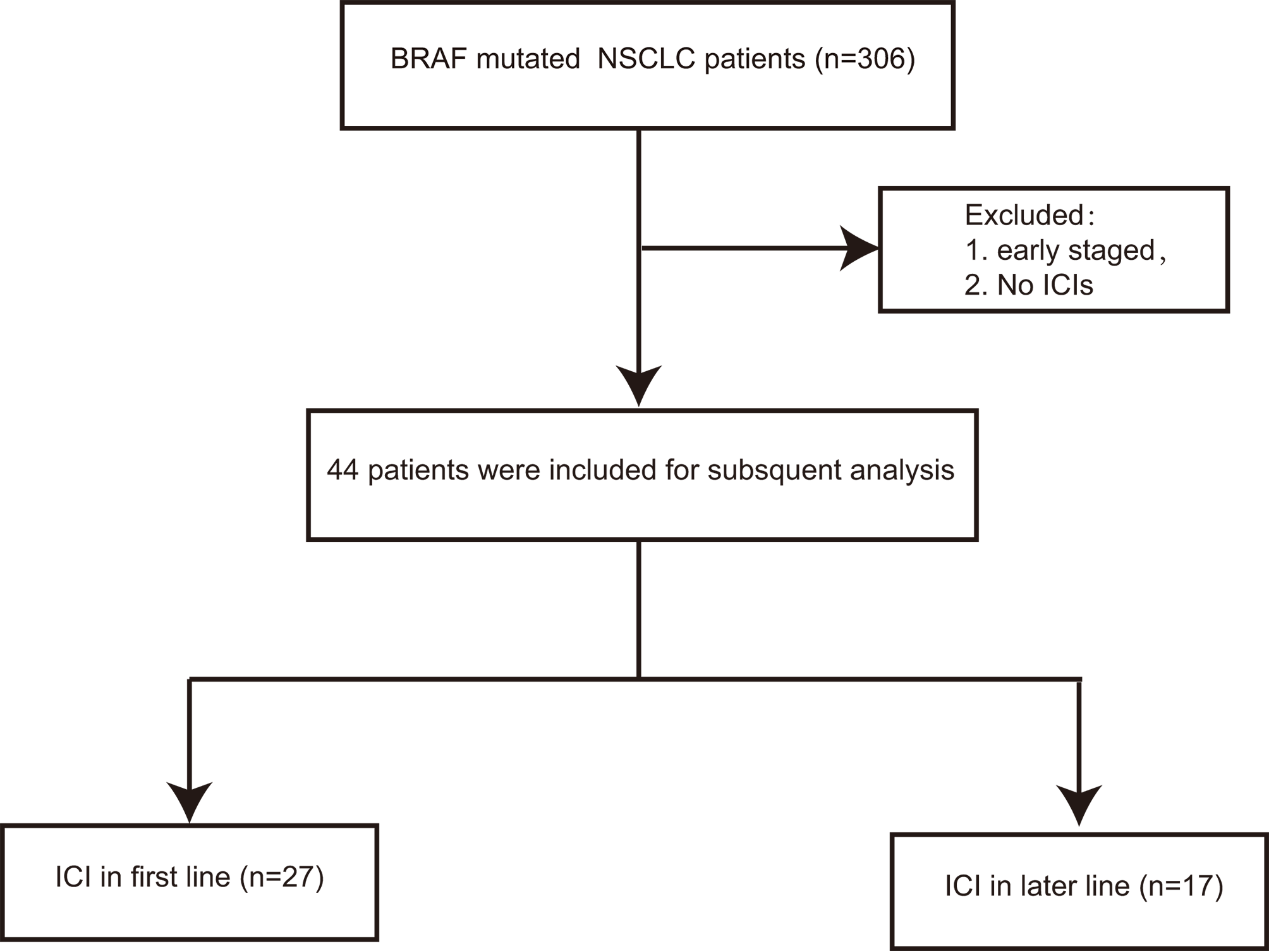


**Supplemental Figure 1** Flow chart of the study

**Supplemental Figure 2**


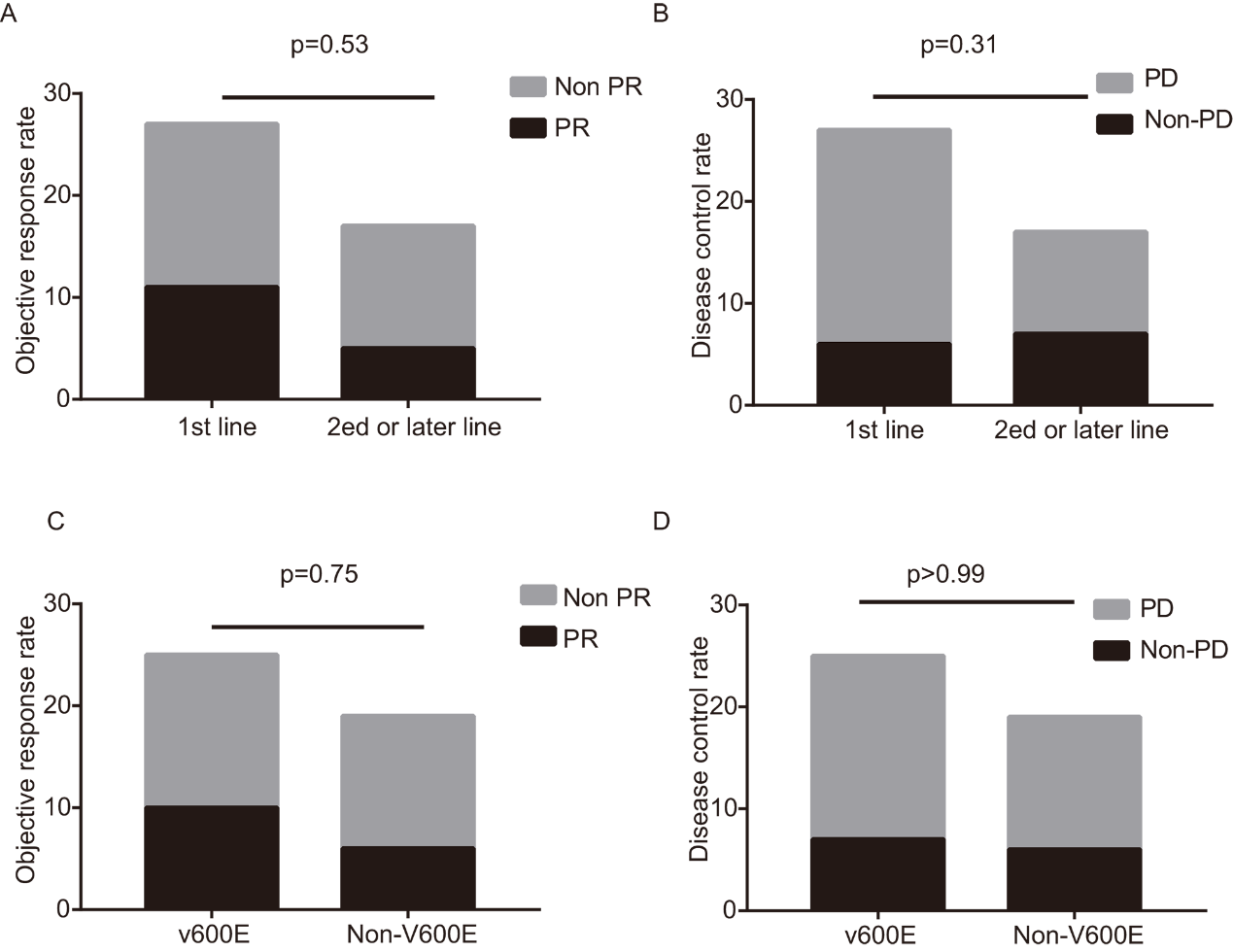


**Supplemental Figure 2.** Response of ICI-treated BRAF-mutated patients in different treatment lines and mutation types. A and B: ORR and DCR for patients in different treatment lines; C and D: ORR and DCR for patients in different BRAF mutation types. ORR, objective response rate, including partial response and complete response; DCR, disease control rate, including stable disease, partial response, and complete response.

**Supplemental Figure 3**


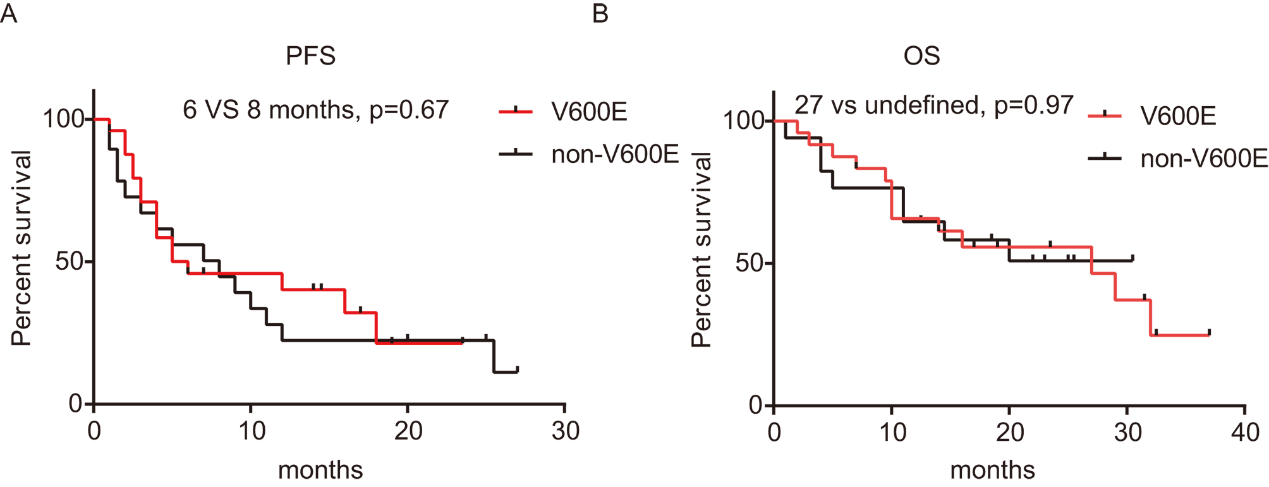


**Supplemental Figure 3.** PFS and OS for patients treated with ICIs based on different mutation types. A and B: PFS and OS of patients with BRAF V600E and non-V600E mutations treated with ICIs. PFS, progression-free survival; OS, overall survival. NE, not evaluable.

**Supplemental Figure 4**


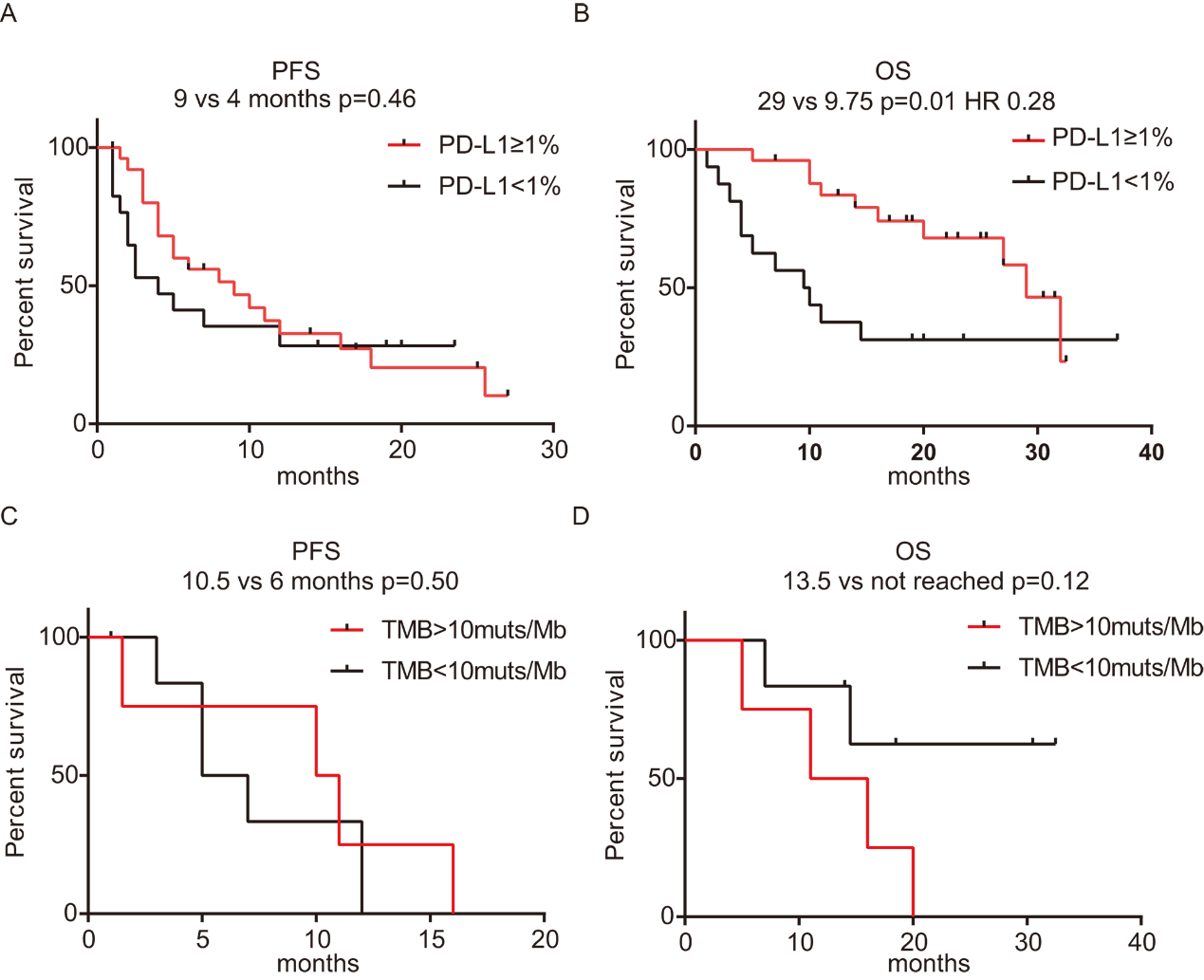


**Supplemental Figure 4.** PFS and OS for patients treated with ICIs stratified based on PD-L1 expression and TMB status. A and B: PFS and OS of ICI-treated patients based on PD-L1 expression; C and D: PFS and OS of ICI-treated patients based on TMB status. PFS, progression-free survival; OS, overall survival. NE, not evaluable; PD-L1, programmed death-ligand 1; TMB, tumor mutation burden.

**Supplemental Table 1** Co-occurring mutations with BRAF mutants

| Co-occurring mutations | Total population |
| --- | --- |
| KRAS | 21/306 (6.9%) |
| HRAS | 1/306 (0.3%) |
| NRAS | 2/306 (0.7%) |
| PI3KCA | 17/306 (5.6%) |
| RB1 | 3/306 (1.0%) |
| TP53 | 20/306 (6.5%) |
| PTEN | 5/306 (1.6%) |
| LRP1B | 8/306 (2.6%) |
| EGFR  Exon 19 deletion  Exon 21 L858R  Exon 18 G719S and exon 20 S768I  Exon 21 L861Q | 3/306 (1.0%)  6/306 (2.0%)  1/306 (0.3%)  1/306 (0.3%) |
| EML4-ALK fusion | 1/306 (0.3%) |
| CCDC6-RET fusion | 1/306 (0.3%) |

**Supplemental Table 2**: Treatment regimens for patients treated with ICIs.

| ID | Regimen | Line | PFS (mo) | Reason for discontinuation | Best response | Mmtation type |
| --- | --- | --- | --- | --- | --- | --- |
| 1 | Pembrolizumab + chemo | 1 | 18 | ongoing | PR | V600E |
| 2 | Camrelizumab + chemo | 1 | 16 | ongoing | PR | V600E |
| 3 | Chemo + Sulglimumab | 1 | 15.5 | SD | SD | Non-V600E |
| 4 | Pembrolizumab + chemo | 1 | 12 | PD | NA | V600E |
| 5 | Camrelizumab + chemo | 1 | 12 | ongoing | PR | Non-V600E |
| 6 | Toripalimab+ chemo | 2 | 12 | PD | PR | Non-V600E |
| 7 | Camrelizumab + chemo | 1 | 11 | PD | NA | Non-V600E |
| 8 | Camrelizumab+chemo | 4 | 10.5 | ongoing | PR | V600E |
| 9 | Camrelizumab +chemo | 1 | 10 | ongoing | SD | Non-V600E |
| 10 | Camrelizumab + chemo | 1 | 10 | PD | PR | Non-V600E |
| 11 | Camrelizumab + chemo | 3 | 7 | PD | SD | Non-V600E |
| 12 | Sintilimab+chemo | 3 | 7 | PD | PR | Non-V600E |
| 13 | Camrelizumab +chemo | 2 | 7 | ongoing | PR | V600E |
| 14 | Camrelizumab + chemo | 1 | 6 | PD | PR | V600E |
| 15 | Camrelizumab + chemo | 3 | 6 | PD | SD | V600E |
| 16 | Camrelizumab + chemo | 3 | 6 | ongoing | SD | V600E |
| 17 | Camrelizumab +chemo | 1 | 6 | ongoing | PR | V600E |
| 18 | Camrelizumab + chemo | 1 | 5 | PD | PR | V600E |
| 19 | Camrelizumab + chemo | 1 | 5 | ongoing | SD | Non-V600E |
| 20 | Camrelizumab + chemo | 1 | 5 | PD | PR | Non-V600E |
| 21 | Camrelizumab + chemo | 3 | 5 | PD | PD | V600E |
| 22 | Camrelizumab + chemo | 1 | 4 | PD | PR | V600E |
| 23 | Camrelizumab + chemo | 1 | 4 | PD | SD | V600E |
| 24 | Camrelizumab + chemo | 1 | 4 | PD | PR | V600E |
| 25 | Camrelizumab + chemo | 3 | 4 | PD | PR | Non-V600E |
| 26 | Camrelizumab + chemo | 1 | 4 | ongoing | SD | V600E |
| 27 | Camrelizumab + chemo | 1 | 3 | PD | PD | V600E |
| 28 | Camrelizumab + chemo | 1 | 3 | PD | PD | V600E |
| 29 | Camrelizumab + chemo | 4 | 3 | ongoing | SD | V600E |
| 30 | Camrelizumab + chemo | 1 | 3 | PD | PD | Non-V600E |
| 31 | Camrelizumab+chemo | 4 | 2.5 | ongoing | SD | V600E |
| 32 | Camrelizumab + chemo | 3 | 2.5 | PD | PD | V600E |
| 33 | Camrelizumab + chemo | 1 | 2 | ongoing | SD | Non-V600E |
| 34 | Camrelizumab+chemo | 2 | 2 | PD | PD | V600E |
| 35 | Camrelizumab + chemo | 1 | 1.5 | PD | PD | Non-V600E |
| 36 | Camrelizumab+chemo | 2 | 1.5 | PD | PD | V600E |
| 37 | Camrelizumab + chemo | 3 | 1 | PD | PD | V600E |
| 38 | Camrelizumab + chemo | 2 | 1 | PD | PD | Non-V600E |
| 39 | Camrelizumab + chemo | 1 | 1 | Ongoing | SD | V600E |
| 40 | Camrelizumab+chemo | 2 | 1 | PD | PD | V600E |
| 41 | Camrelizumab + chemo | 1 | 1 | Ongoing | SD | Non-V600E |
| 42 | Pembrolizumab+chemo | 1 | 9 | PD | SD | Non-V600E |
| 43 | Pembrolizumab + chemo | 1 | 14 | PR | PR | V600E |
| 44 | Camrelizumab + chemo | 1 | 7 | PD | SD | V600E |

chemo, chemotherapy; PFS, progression free survival; mo, months; PD, progressive disease; PR, partial response; SD, stable disease; NA, not available.

**Supplemental Table 3** PD-L1 expression levels of 163 patients with BRAF mutation

| Patients No. | PDL1 level (%) |
| --- | --- |
| 1 | <1 |
| 2 | 0 |
| 3 | <1 |
| 4 | Negtive |
| 5 | 0 |
| 6 | 0 |
| 7 | Negtive |
| 8 | 0 |
| 9 | 0 |
| 10 | 0 |
| 11 | 0 |
| 12 | Negtive |
| 13 | 0 |
| 14 | 0 |
| 15 | 0 |
| 16 | 0 |
| 17 | 0 |
| 18 | 0 |
| 19 | 0 |
| 20 | <1 |
| 21 | <1 |
| 22 | 0 |
| 23 | 0 |
| 24 | 0 |
| 25 | 0 |
| 26 | 0 |
| 27 | 0 |
| 28 | 0 |
| 29 | 0 |
| 30 | 0 |
| 31 | 0 |
| 32 | Negtive |
| 33 | 0/40 |
| 34 | 0 |
| 35 | 0 |
| 36 | 0 |
| 37 | 0 |
| 38 | 0 |
| 39 | <1 |
| 40 | 0 |
| 41 | Negtive |
| 42 | 0 |
| 43 | 0 |
| 44 | 0 |
| 45 | 0 |
| 46 | 0 |
| 47 | 0 |
| 48 | Negtive |
| 49 | 0 |
| 50 | <1 |
| 51 | 0 |
| 52 | <1 |
| 53 | 0 |
| 54 | Negtive |
| 55 | Negtive |
| 56 | 0 |
| 57 | <1 |
| 58 | 0 |
| 59 | 0 |
| 60 | <1 |
| 61 | Negtive |
| 62 | 0 |
| 63 | 0 |
| 64 | 0 |
| 65 | 0 |
| 66 | <1 |
| 67 | 0 |
| 68 | 0 |
| 69 | <1 |
| 70 | 0 |
| 71 | 0 |
| 72 | 0 |
| 73 | <1 |
| 74 | 0 |
| 75 | 0 |
| 76 | 0 |
| 77 | 0 |
| 78 | 0 |
| 79 | 0 |
| 80 | 0 |
| 81 | 0 |
| 82 | 0 |
| 83 | Negtive |
| 84 | Negtive |
| 85 | 0 |
| 86 | <1 |
| 87 | 0 |
| 88 | 0 |
| 89 | <1 |
| 90 | 20 |
| 91 | 15 |
| 92 | 20 |
| 93 | 20 |
| 94 | 30 |
| 95 | 40 |
| 96 | 10 |
| 97 | 3 |
| 98 | 40 |
| 99 | 1 |
| 100 | 5 |
| 101 | 1 |
| 102 | 3 |
| 103 | 5 |
| 104 | 10 |
| 105 | 1 |
| 106 | 2 |
| 107 | 10 |
| 108 | 20 |
| 109 | 5 |
| 110 | 10 |
| 111 | 10 |
| 112 | 5 |
| 113 | 5 |
| 114 | 30 |
| 115 | 1 |
| 116 | 10 |
| 117 | 10 |
| 118 | 20 |
| 119 | 30 |
| 120 | 30 |
| 121 | 5 |
| 122 | 2 |
| 123 | 3 |
| 124 | 5 |
| 125 | 40 |
| 126 | 5 |
| 127 | 40 |
| 128 | 5 |
| 129 | 3 |
| 130 | 1 |
| 131 | 20 |
| 132 | 20 |
| 133 | 5 |
| 134 | 20 |
| 135 | 5 |
| 136 | 5 |
| 137 | 1 |
| 138 | 1 |
| 139 | 40 |
| 140 | 25 |
| 141 | 5 |
| 142 | 60 |
| 143 | 90 |
| 144 | 90 |
| 145 | 80 |
| 146 | 70 |
| 147 | >90 |
| 148 | 80 |
| 149 | 70 |
| 150 | 80 |
| 151 | 90 |
| 152 | 50 |
| 153 | 100 |
| 154 | 90 |
| 155 | 60 |
| 156 | 95 |
| 157 | 60 |
| 158 | 80 |
| 159 | 80 |
| 160 | 60 |
| 161 | 70 |
| 162 | 70 |
| 163 | 90 |

**Supplemental table 4** Subsequent anticancer therapy

| Subsequent therapies n (%) | Chemo-immunotherapy in first line (n=27) |
| --- | --- |
| Patients receiving any subsequent therapy, n (%) | 21 (77.8) |
| BRAF TKIs | 2 (7.4) |
| Immunotherapy based regimens | 8 (29.6) |
| Anti-anigogenisis agents | 2 (7.4) |
| chemotherapy | 7 (25.9) |
| Other systemic therapies | 2 (7.4) |

**Supplemental Table 5** Best clinical benefit in patients with BRAF mutations treated with ICI combined therapies

| Best response | All patients n% | ICI in 1^st^ line | ICI in 2ed or later line |
| --- | --- | --- | --- |
| CR | 0 | 0 | 0 |
| PR | 16 (36.4%) | 11 (40.7%) | 5 (29.4%) |
| SD | 15 (34.1%) | 10 (37.0%) | 5 (29.4%) |
| PD | 13 (29.5%) | 6 (22.2%) | 7 (41.2%) |
| ORR | 16 (36.3%; 95%CI, 21.6%-51.2%) | 11 (40.7%; 95% CI: -20.9%-60.5%) | 5 (29.4%;95% CI: 5.3%-53.6%) |
| DCR | 31 (70.5%, 95%CI: 56.4%-84.5%) | 21 (77.8%;95% CI -61.0%-94.5%) | 10 (58.8%; 95% CI 32.7%-84.9%) |

**Supplemental Table 6** adverse events of any cause in the ICI combined regimens-treated Population

| event | treated population (30 patients have documented side effects) | |
| --- | --- | --- |
|  | Any grade | Grade 3, 4, 5 |
| Gastrointestinal effects | 6/30 (20%) | 1/30 (3.3%) |
| Skin effects | 7/30 (23.3%) | 0 |
| anemia | 11/30 (36.7%) | 0 |
| neutropenia | 5/30 (16.6%) | 0 |
| thrombocytopenia | 4/30 (14.3%) | 1/30 (3.3%) |
| Interstitial Lung Disease | 3/30 (10.0%) | 1/30 (3.3%) |
| liver injury | 9/30 (30.0%) | 0 |
| hypothyroidism | 4/30 (14.3%) | 0 |
